# Disruption of the *Eng18B* ENGase Gene in the Fungal Biocontrol Agent *Trichoderma atroviride* Affects Growth, Conidiation and Antagonistic Ability

**DOI:** 10.1371/journal.pone.0036152

**Published:** 2012-05-07

**Authors:** Mukesh K. Dubey, Wimal Ubhayasekera, Mats Sandgren, Dan Funck Jensen, Magnus Karlsson

**Affiliations:** 1 Department of Forest Mycology and Pathology, Uppsala BioCenter, Swedish University of Agricultural Sciences, Uppsala, Sweden; 2 MAX-lab, Lund University, Lund, Sweden; 3 Institute of Medicinal Chemistry, University of Copenhagen, Copenhagen, Denmark; 4 Department of Molecular Biology, Swedish University of Agricultural Sciences, Uppsala, Sweden; Seoul National University, Republic of Korea

## Abstract

The recently identified phylogenetic subgroup B5 of fungal glycoside hydrolase family 18 genes encodes enzymes with mannosyl glycoprotein endo-*N*-acetyl-β-D-glucosaminidase (ENGase)-type activity. Intracellular ENGase activity is associated with the endoplasmic reticulum associated protein degradation pathway (ERAD) of misfolded glycoproteins, although the biological relevance in filamentous fungi is not known. *Trichoderma atroviride* is a mycoparasitic fungus that is used for biological control of plant pathogenic fungi. The present work is a functional study of the *T. atroviride* B5-group gene *Eng18B*, with emphasis on its role in fungal growth and antagonism. A homology model of *T. atroviride* Eng18B structure predicts a typical glycoside hydrolase family 18 (αβ)_8_ barrel architecture. Gene expression analysis shows that *Eng18B* is induced in dual cultures with the fungal plant pathogens *Botrytis cinerea* and *Rhizoctonia solani*, although a basal expression is observed in all growth conditions tested. *Eng18B* disruption strains had significantly reduced growth rates but higher conidiation rates compared to the wild-type strain. However, growth rates on abiotic stress media were significantly higher in *Eng18B* disruption strains compared to the wild-type strain. No difference in spore germination, germ-tube morphology or in hyphal branching was detected. Disruption strains produced less biomass in liquid cultures than the wild-type strain when grown with chitin as the sole carbon source. In addition, we determined that Eng18B is required for the antagonistic ability of *T. atroviride* against the grey mould fungus *B. cinerea* in dual cultures and that this reduction in antagonistic ability is partly connected to a secreted factor. The phenotypes were recovered by re-introduction of an intact *Eng18B* gene fragment in mutant strains. A putative role of Eng18B ENGase activity in the endoplasmic reticulum associated protein degradation pathway of endogenous glycoproteins in *T. atroviride* is discussed in relation to the observed phenotypes.

## Introduction

Enzymes with mannosyl glycoprotein endo-*N*-acetyl-β-D-glucosaminidase (ENGase)-type activity (EC.3.2.1.96) are found in glycoside hydrolase (GH) families 18, 73 and 85 [1]. ENGases are deglycosylation enzymes, which act on the di-*N*-acetylchitobiosyl part of *N*-glycosidically linked oligosaccharides [2]. Biochemically characterized fungal ENGase sequences are reported from the GH family 85 Endo M from *Mucor hiemalis* [3], and recently from the GH family 18 members *Trichoderma reesei* Eng18A, (also referred to as Endo T, [4]), and *Flammulina velutipes* Endo FV [5]. Both *T. reesei* Eng18A and *F. velutipes* Endo FV belongs phylogenetically to the fungal GH family 18 subgroup B5 [6]. Phylogenetic relationships with other fungal GH family 18 subgroups that contain biochemically characterized chitinases (EC.3.2.1.14) suggest a single neofunctionalization event that resulted in evolution of enzymes with ENGase activity from a chitinase ancestor [7].

There are two GH family 18 subgroup B5 ENGase members in *T. reesei*, Eng18A and Eng18B [4], with orthologs in *T. atroviride* and *T. virens* [8]. *T. reesei* Eng18A contains a signal peptide for secretion, and is purified from *T. reesei* extracellular growth medium [4]. *T. reesei* Eng18A may thus be responsible for postsecretorial modifications of glycan structures on endogenous *T. reesei* glycoproteins such as cellulases, or participate in hydrolysis of the oligosaccharide-protective coat of foreign glycoproteins to generate nutrients.

The second *T. reesei* B5 ENGase member, Eng18B, is devoid of a signal peptide and is therefore predicted to have an intracellular localization. Intracellular ENGase activity is reported from both animals and plants where it is an integrated part of the endoplasmic reticulum (ER) associated protein degradation pathway (ERAD) [9], [10]. Misfolded glycoproteins are identified inside the ER, and prevented to enter the Golgi for further secretion. Misfolded glycoproteins are eventually degraded by the ERAD-pathway, which involves translocation from the ER to the cytosol and subsequent degradation by the 26S proteasome. Prior to proteolysis, *N*-glycan carbohydrate chains are removed by peptide:*N*-glycanases (PNGases, EC.3.5.1.52) to generate free oligosaccharides with an intact di-*N*-acetylchitobiose moiety at their reducing termini (fOS-GN2), followed by further cleavage of the di-*N*-acetylchitobiose moiety by ENGases that results in free oligosaccharides with a single *N*-acetylglucosamine (GlcNAc) at their reducing termini (fOS-GN1). Finally, α-mannosidases (EC.3.2.1.24) acts on the fOS before transport into the lysosome for final degradation [9], [10].

*Saccharomyces cerevisiae* yeast strains that are deficient in fOS production [11] and degradation [12] do not display any growth phenotypes. However, the situation is more complicated in filamentous fungi. Disruption of the cytosolic PNGase gene *png-1* in the filamentous fungus *Neurospora crassa* manifests in a swollen-tip phenotype and reduced cell wall integrity [13], [14]. However, the *N. crassa* PNG-1 protein contain several amino acid substitutions that results in lack of enzymatic activity, implying an unknown function of PNG-1 independent from the PNGase enzymatic activity [14]. These substitutions are present in several PNG-1 orthologs in the fungal kingdom, raising questions concerning the mechanisms of ERAD-pathway dependent glycoprotein deglycosylation in filamentous fungi. In addition, no studies are available on the importance of the recently discovered fungal GH family 18 ENGases for fungal growth and development. Therefore we generated a disruption mutant of *T. atroviride* Eng18B, orthologous to *T. reesei* Eng18B, and analysed resulting defects in growth and development. *T. atroviride* is a mycoparasitic species that attack and kill other fungi, and it is therefore commercially used as a biological control agent against plant pathogenic fungi in agricultural and horticultural production systems [15].

In this study we show that expression of *T. atroviride Eng18B* is induced in dual cultures with the fungal plant pathogens *Botrytis cinerea* and *Rhizoctonia solani*, although a basal expression is observed in all growth conditions tested. By generating Δ*Eng18B* disruption mutants we show that Eng18B is involved in vegetative growth, tolerance to abiotic stress and conidiation. In addition, disruption of *T. atroviride* Eng18B results in a reduced ability to utilize chitin in liquid cultures and in reduced antagonistic ability towards *B. cinerea* but not towards other fungi or oomycetes.

## Materials and Methods

### Sequence Analysis

The *T. atroviride* genome sequence v.2 (http://genome.jgi-psf.org/Triat2/Triat2.home.html) was used for gene sequence retrieval. Analyses for conserved domains were performed using the SMART protein analysis tool [16], InterProScan [17] and Conserved Domain Search [18]. Signal P version 3.0 [19] was used to search for signal peptide cleavage sites, TMHMM version 2.0 [20] was used to search for transmembrane helices, and the big-PI Fungal Predictor program [21] was used to search for GPI-anchor sequences.

Partial *Eng18B* sequences from seven *Trichoderma* species (Table 1) were generated by PCR amplification and sequencing using primers P21–P26 listed in Table S1. Sequences were submitted to GenBank [22] with accession numbers JF300121-JF300127. Regions of low amino acid conservation between Eng18B *Trichoderma* orthologs was identified by Reverse Conservation Analysis (RCA) as described previously [23].

**Table 1:** *Trichoderma* species used for *Eng18B* sequencing, and translated amino acid positions used for Reverse Conservation Analysis.

Species^a^	CBS strain number	Amino acid positions^b^	Sequence length (aa)
*T*. *atroviride^c^*	-	11→337	327
*T*. *reesei^c^*	-	7→329	323
*T*. *virens^c^*	-	8→327	320
*T*. *asperellum*	433.97	11→265	255
*T*. *citrinoviride*	258.85	11→259	249
*T*. *harzianum*	102174	87→270	184
*T*. *piluliferum*	224.84	87→269	183
*T*. *brevicompactum*	109720	94→266	173
*T. tomentosum*	349.93	11→181	171
*T. croceum*	337.93	89→265	177

a Species identification based on internal transcribed spacer (ITS) sequencing and TrichOKey identification as described previously [8], ^b^Translated amino acid sequence positions used for Reverse Conservation Analysis is given using *T*. *atroviride* Eng18B as reference, ^c^Sequences retrieved from genome sequences; *T. atroviride* protein ID 302173, *T. reesei* protein ID 121335, *T. virens* protein ID 92008.

### Homology Modelling

To date the only available fungal GH family 18 ENGase structure deposited in the Protein Data Bank (PDB) [24] is for Eng18A from *T. reesei*. The amino acid sequences of *T. atroviride* Eng18B (protein ID 302173) and *T. reesei* Eng18A (Uniprot protein ID C4RA89) were aligned using Clustal W [25]. The homology model of the catalytic module of *T. atroviride* Eng18B was built based on the structure of Eng18A from *T. reesei* (PDB entry 4AC1; which has a sequence identity of 44% to *T. atroviride*) using the program Modeller version 9.10 [26]. The *T. atroviride* Eng18B structure homology model will be available upon request to the authors. The structure model figure was prepared using the program PyMol [27].

### Fungal Strains and Culture Conditions

*T. atroviride* strain IMI206040 (WT) and mutants derived from it, *Aspergillus nidulans* strain A4, *B. cinerea* strain B05.10, *Fusarium graminearum* strain PH1, *Heterobasidion occidentale* strain 122-12, *Phanerochaete chrysosporium* strain RB75, *Phytophthora niederhauseri* strain P1017 and *R. solani* strain SA1 were maintained on potato dextrose agar (PDA) (Oxoid, Cambridge, UK) medium at 25°C in darkness, while *N. crassa* strain 2489 was maintained on Vogels media [28]. SMS medium supplemented with 1% glucose was used for gene expression and phenotypic screening unless otherwise specified. The composition of SMS medium was (in g/L): KH_2_PO_4_, 2; (NH_4_)_2_SO_4_, 1.4; Mg_2_SO_4_×7H_2_O, 0.3; CaCl_2_×2H_2_O, 0.3; FeSO_4_×7H_2_O, 0.005; ZnSO_4_×7H_2_O, 0.002; MnSO_4_×H_2_O, 0.002. Culture medium for different carbon sources were prepared by substituting 1% glucose in SMS medium with colloidal chitin (1%), R. solani cell walls (RsCW) (1%), glucose (5%) or GlcNAc (1 mM). Starvation for carbon (C lim), nitrogen (N lim) and carbon + nitrogen (C+N lim) was induced by replacing 1% glucose with 0.1%, 1.4 g/L (NH_4_)_2_SO_4_ with 0.14 g/L and 1% glucose +1.4 g/L (NH_4_)_2_SO_4_ with 0.1%+0.14 g/L, respectively. The agar surface in agar plates was covered with cellophane to facilitate harvesting of mycelium. *T. atroviride* mycelia for submerged liquid cultures were pre-cultivated in 100 ml of SMS on rotary shaker (200 rpm) at 25°C in darkness for 48 h, followed by harvesting by filtering through Miracloth, washed with sterile distilled water and transferred to new flasks containing 50 ml of fresh SMS medium containing different nutrient regimes as described above. Colloidal chitin was prepared from crab-shell colloidal chitin (Sigma-Aldrich, St. Louis, MO) as described previously [29]. R. solani cell wall material was prepared using the method described by Inglis and Kawchuk [30] with minor modifications.

### Gene Expression Analysis

For *T. atroviride Eng18B* expression analysis, shake flask cultures were prepared as described above and mycelia were harvested 24 h post inoculation using vacuum filtration, washed three times in distilled sterile water, frozen in liquid nitrogen and stored at -80°C. For plate confrontation assays, *T. atroviride* and *B. cinerea* or *R. solani* were inoculated on opposite sides of a 9 cm PDA plate covered with cellophane. Mycelia from the growing front (7–10 mm) of *T*. *atroviride* 24 h after contact were harvested and immediately frozen in liquid nitrogen and stored at −80°C. *T*. *atroviride* confronted with itself was used as control treatment. After grinding mycelia in liquid nitrogen, total RNA was extracted using 3% hexadecyl-tri-methyl-ammonium bromide (CTAB) detergent and phenol-chloroform purification, followed by NaOAc/ethanol purification and selective precipitation of RNA with 8 M LiCl. RNA was treated with RNAse free DNaseI (Fermentas, St. Leon-Rot, Germany) and concentrations were determined spectrophotometrically using NanoDrop (Thermo Scientific, Wilmington, DE).

RevertAid premium reverse transcriptase (Fermentas, St. Leon-Rot, Germany) was used for cDNA-synthesis, while transcript levels were quantified by quantitative PCR (qPCR) using the SYBR Green PCR Master Mix (Applied Biosystems, Foster City, CA). Each 20 µl qPCR reaction contained cDNA template, 150 nM of each primer (P17/P18 or P19/P20, Table S1), 1× SYBR green fluorescent dye and other components according to the SYBR Green PCR Master Mix protocol and performed in an iQ5 qPCR System (Bio-Rad, Hercules, CA) including melt curve analysis. Relative expression levels for *T. atroviride Eng18B* in relation to actin (protein ID 297070) expression were calculated from the Ct values and the primer amplification efficiencies by using the formula described by Pfaffl [31]. QPCR reactions were performed in three biological replicates, each based on two technical replicates.

### Construction of *T. atroviride Eng18B* Disruption and Complementation Vectors

Genomic DNA was isolated using a CTAB-based method [32]. *T. atroviride Eng18B* flanking regions were PCR amplified using Phusion DNA polymerase (Finnzymes, Vantaa, Finland) and primers P1/P2 and P5/P6 (Table S1, Fig. S1A), while primers P3/P4 was used to PCR amplify the hygromycin resistance gene (*hph*) cassette from the pCT74 vector [33]. Gateway® cloning technology (Invitrogen, Carlsbad, CA) and destination vector pPm43GW [34] was used to generate the disruption vector pPm43GW-Eng18B-ko. The *Eng18B* full-length sequence was PCR amplified with primers P29/P30, while primers P31/P32 was used to PCR amplify the nourseothricin resistance gene (*nat1*) cassette from the pD-NAT1 vector [35]. For technical reasons a 311 bp, non-coding fragment (contig 15, position 293545 to 293855) was amplified with primers P15/P16, and used together with the *Eng18B* and *nat1* DNA fragments to generate the complementation vector pPm43GW-Eng18B-comp by Gateway® cloning technology (Invitrogen, Carlsbad, CA).

### Agrobacterium Tumefaciens Mediated Transformation

The disruption (pPm43GW-Eng18B-ko) and complementation (pPm43GW-Eng18B-comp) vectors were transformed into *Agrobacterium tumefaciens* strain AGL-1 following a freeze thaw procedure [36] and positive clones were selected on YEP (for 1 L; 10 g yeast extract, 10 g bacto peptone, 5 g NaCl; pH adjusted to 7.0) plates containing 35 µg/mL rifampicin (Sigma-Aldrich, St. Louis, MO) and 100 µg/mL spectinomycin (Sigma-Aldrich, St. Louis, MO). *A. tumefaciens*-mediated transformation (ATMT) of *T*. *atroviride* was performed based on a previous protocol for *T*. *harzianum* [37]. Mitotically stable *Eng18B* disruption (Δ*Eng18B*) and Δ*Eng18B*-*Eng18B*-complemented (Δ*Eng18B*+) transformants were purified by two rounds of single spore isolation.

### Validation of Transformants

Homologous integration of the disruption cassette was evaluated by PCR using primers specific to the *hph* gene in combination with primers specific to sequences flanking the deletion construct (P3/P12, P4/P11). Reverse transcriptase (RT)-PCR was conducted on WT, Δ*Eng18B* and Δ*Eng18B*+ strains using primers specific for *hph*, *nat1*, *Eng18B* and translation elongation factor *tef1* (P13/P14, P33/P34, P19/P20 and P7/P8, Table S1). A mitotically stable Δ*Eng18B*+ complementation strain was included in all phenotype analyses to exclude the possibility of phenotypes that derive from ectopic insertions.

### Analyses of Morphology, Growth Rate, Conidiation and Biomass Production

Colony morphology and growth diameter were recorded in triplicates daily. Conidiation was determined in triplicates as described before [38] using a Bright-Line haemocytometer (Sigma-Aldrich, St. Louis, MO). Biomass production in 25 ml liquid cultures was analysed in triplicates by determining mycelial dry weight after incubation at 25°C in darkness for 3 days under constant shaking condition (100 rpm). For liquid cultures containing colloidal chitin and *R. solani* cell wall material, spectrophotometrically determined protein content was used as a measure of biomass production as described previously for *T. atroviride* [38]. Abiotic stress tolerance was evaluated in triplicates by measuring colony diameter after 7 days of growth on PDA plates containing 1 M NaCl or 0.025% SDS.

### Enzyme Activity Assays

β-*N*-acetylhexosaminidase (NAGase, EC.3.2.1.52) and endochitinase activity was measured in triplicates using (GlcNAc)_1&3_ conjugated to 4-methylumbelliferyl (4-MU) as substrates (Sigma-Aldrich, St. Louis, MO), respectively. An 85 µl sample was mixed with 15 µl of substrate (35 µM) in a 96-well micro-plate and incubated at room temperature for 20 min, followed by addition of 100 µl of 1 M glycine buffer, pH 10.6. Fluorescence of released 4-MU was determined by using a luminescence spectrometer, model LS50B (Perkin Elmer, Waltham, MA) at Ex360/Em455. For extracellular activity, culture filtrates from 5 days of fungal growth in SMS supplemented with 1% colloidal chitin or 1% *R. solani* cell walls was used. In addition, harvested mycelia were ground in liquid nitrogen and suspended in 1 ml of TE buffer (100 mM Tris pH 8.0, 1 mM EDTA pH 8.0), centrifuged at 13000 rpm, 4°C for 10 min and the supernatant was used to assay intracellular enzyme activity.

ENGase deglycosylation activity was measured in triplicates in culture filtrate and mycelial fractions using an RNase B SDS polyacrylamide gel mobility shift assay [4]. *T*. *atroviride* was grown in dextrose broth (D-glucose 20 g/L, Tryptone 5 g/L and peptone 5 g/L) medium for 48 h at 25°C. The mycelial fraction was prepared by grinding harvested and washed mycelia in 1 ml of 100 mM sodium acetate buffer, pH 5.0, followed by centrifugation for 10 min at 13000 rpm at 4°C, and collection of the supernatant. To monitor ENGase activity, a 40 µl sample was incubated with 10 µl of the highly glycolylated RNAse B (Sigma-Aldrich, St. Louis, MO) at a concentration of 10 mg/ml dissolved in 100 mM sodium acetate buffer, pH 5.0, [4] for 24 h at room temperature. Deglycosylation of RNAse B was monitored by mobility shift of bands on 4–20% SDS-PAGE gel (Bio-Rad, Hercules, CA) after staining with Coomassie Brilliant Blue. RNase B incubated with dextrose broth medium was used as control.

### Antagonism Test

Antagonistic behaviour of *T*. *atroviride* towards other fungi was tested in triplicates using an *in vitro* plate confrontation assay on SMS agar plates. Secreted factors were assayed by growing *T. atroviride* on SMS agar plates covered with cellophane at 25°C in darkness. The cellophane was removed when *T. atroviride* covered the plates, followed by inoculation with *B. cinerea* and growth was measured daily in three replicates. In addition, *B. cinerea* was grown on SMS agar plates covered with cellophane at 25°C in darkness, followed by removal of the cellophane and inoculation with *T. atroviride*. Linear growth was recorded daily in three replicates.

### Microscopy Analysis

Conidial germination in PDB medium was observed after 20 h using a Zeiss Axioplan microscope (Thornwood, NY) equipped with Leica application suite version 3.6.0 and images were taken using a Leica DFC295 digital camera (Wetzlar, Germany). Fungal material was stained with 0.1% calcofluor-white stain (Sigma-Aldrich, St. Louis, MO) diluted in PBS. Stained material was examined using a Leica DM5500B microscope (Wetzlar, Germany) with DAPI filters.

### Statistical Analysis

Analysis of variance (ANOVA) was performed on gene expression and phenotype data using a General Linear Model approach implemented in Statistica version 10 (StatSoft, Tulsa, OK). Pairwise comparisons were made using the Tukey-Kramer method at the 95% significance level. In addition, gene expression data were analysed by Student’s *t*-test implemented in Statistica.

## Results

### Bioinformatic Analysis and Homology Modelling of *T. atroviride* Eng18B

Protein ID 302173 was retrieved from the *T. atroviride* genome sequence v.2 and named Eng18B. In addition, the second B5-group *T. atroviride* member, protein ID 217415, was retrieved and named Eng18A. The *Eng18B* transcript was 1377 bp long and contained a coding region of 1014 bp without any introns. The translated Eng18B 337 amino acid (aa) sequence was analysed for conserved domains using SMART which identified a single GH family 18 module between aa positions 12–269 (Pfam00704), including a putative catalytic motif DGLDLDVE (aa pos. 127–134) and a putative substrate binding site SLGG (aa pos. 138–141). This structure was also confirmed by InterProScan and Conserved Domain Search analyses. No N-terminal secretion signal peptide, transmembrane domains, or C-terminal GPI-anchor signal were found from analyses using SignalP, TMHMM, and big-PI Fungal Predictor respectively. Manual inspection identified one putative glycosylation site NLS (aa pos. 174–176) that conformed to the conserved N-X-S/T sequence where N is the acceptor for the oligosaccharide structure [39]. The GH family 18 module of *T. atroviride* Eng18B displayed 86% identical residues to the *T. reesei* ortholog (Eng18B) and 50% identity to *T. atroviride* Eng18A (orthologous to *T. reesei* Eng18A (also known as Endo T)).

**Figure 1:**
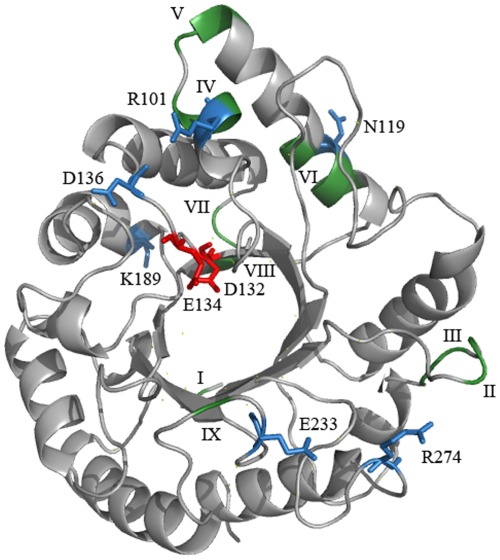
*Trichoderma atroviride* Eng18B homology model. Ribbon diagrams of the catalytic module of Eng18B, based on the structure of *T. reesei* Eng18A (PDB entry 4AC1) showing the conserved catalytically important residues in red; variable regions from reverse conservation analysis (Wmeans) in green; and highly variable amino acid positions with Sscore ≥3 in blue. Variable regions are marked in Roman numerals from N- to C- termini.

Homology modelling of *T. atroviride* Eng18B revealed that the catalytic module of the enzyme has a (αβ)_8_ barrel architecture, which is indicative of a GH family 18 enzyme. The residues in the enzyme that are considered to be important for the catalytic activity of the enzyme (Asp132, Glu134 and Tyr194) were completely conserved between the two fungal ENGase enzymes, and most the amino acids considered to be important for substrate recognition were highly conserved between the two enzymes (Fig. 1). According to the RCA analysis, high amino acid diversity between Eng18B orthologs was distributed amongst twelve regions (I-XII, Fig. 1) in the protein. Region X corresponded to a putative linker region, while regions III and IX bordered the catalytic cleft and may influence the structure of the substrate-binding cleft of the enzyme (Fig. 1). All other eight regions were predicted to be distant from the catalytic cleft.

### Expression of *T. atroviride Eng18B* is Induced in Antagonistic Interactions

**Figure 2:**
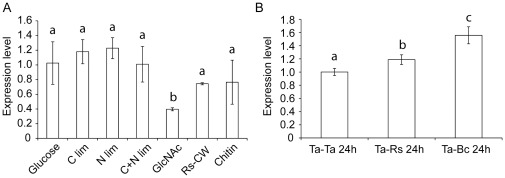
Expression analysis of the *T*. *atroviride Eng18B* gene. Total RNA was extracted from *T*. *atroviride* mycelia after 24 h of incubation in submerged shake flask cultures at 25°C in darkness representing different nutritional/stress and mycoparasitic conditions. (A) *T*. *atroviride Eng18B* expression in glucose, C limitation, N limitation, C+N limitation, *N*-acetylglucosamine (GlcNAc), *R. solani* cell wall material (RsCW) and colloidal chitin mediums. (B) *Eng18B* expression 24 h after contact with *R. solani* (Ta-Rs24h) or *B. cinerea* (Ta-Bc24h). *T*. *atroviride* confronted with itself was used as control (Ta-Ta24h). Relative expression levels for *Eng18B* in relation to actin expression were calculated from the Ct values and the primer amplification efficiencies by using the formula described by Pfaffl [42]. Error bars represent standard deviation based on three biological replicates. Experiments in panel A and B were analysed separately, different letters indicate statistically significant differences (*P*≤0.05) within experiments.

Quantitative PCR was used to analyse gene expression patterns of *T*. *atroviride Eng18B* under conditions relevant for ENGase induction and mycoparasitism. Experiment A was performed in liquid SMS cultures supplemented with either glucose, GlcNAc, chitin, *R. solani* cell walls, or representing carbon limitation, nitrogen limitation or the combination of the two. Expression of *T*. *atroviride Eng18B* was measured after 24 h of growth and showed a significant (*P* = 0.023) 2.6-fold repression in SMS medium containing GlcNAc as a sole carbon source compared to the glucose control (Fig. 2A). Experiment B was performed as dual cultures between *T. atroviride* and *R. solani* or *B. cinerea* on PDA agar plates, with a *T. atroviride* self-self interaction as control. The transcription of *T*. *atroviride Eng18B* was found to be significantly (*P*≤0.043) induced 24 h after contact with either species (1.2 and 1.6-fold, respectively. Fig. 2B). Interaction with *B. cinerea* induced a 1.3 fold stronger response (*P*<0.001) compared with *R. solani*. An additional observation was that a basal expression of the *Eng18B* gene was observed in all tested culture conditions.

### Disruption of *Eng18B* in *T*. *atroviride*

A disruption vector pPm43GW-Eng18B-ko was constructed and introduced in *A*. *tumefaciens* to replace the *Eng18B* gene in *T*. *atroviride* using ATMT. Gene replacement in 10 randomly selected, hygromycin-resistant transformants were confirmed by PCR using primer pair P3/P4 for *hph* cassette, P11/P4 for upstream and P3/P12 for downstream amplification. Expected size of PCR fragment was amplified in all 10 mutants, while no amplification was observed in WT (Fig. S1B, C and D, respectively). To verify the complete replacement of the *Eng18B* gene, PCR amplification using primer pair P11/P12 flanking the deletion construct, was performed and generated the expected 3.8 kb and 4.3 kb PCR products from WT and mutant strains, respectively (Fig. S1E).

RT-PCR on cDNA from four randomly selected positive disruption mutants along with the WT using the *Eng18B*-specific primer pair P19/P20 demonstrated the lack of *Eng18B* transcripts in any of the mutant strains (Fig. S1F). An RT-PCR product of 170 bp from the *hph* gene was obtained from the four mutants using primers P13/P14, whereas no amplification product was found in the WT strain (Fig. S1F). Amplification of a *tef1* fragment from both WT and mutant *T*. *atroviride* strains demonstrated that the cDNA was of sufficient quality (Fig. S1F).

Successful integration of the pPm43GW-Eng18B-comp *Eng18B* complementation cassette in six independent, mitotically stable Δ*Eng18B* strains was confirmed by PCR amplification of the *nat1* selection marker using primers P33/P34 from genomic DNA (Fig. S1G). RT-PCR on cDNA from three randomly selected *nat1* positive Δ*Eng18B*+ strains using the *Eng18B*-specific primer pair P19/P20 demonstrated restored *Eng18B* transcription, while no transcripts were detected in the parental Δ*Eng18B* deletion strains (Fig. S1H).

### Disruption of *T*. *atroviride Eng18B* Results in Colony Morphology Change and High Rates of Conidiation

**Figure 3:**
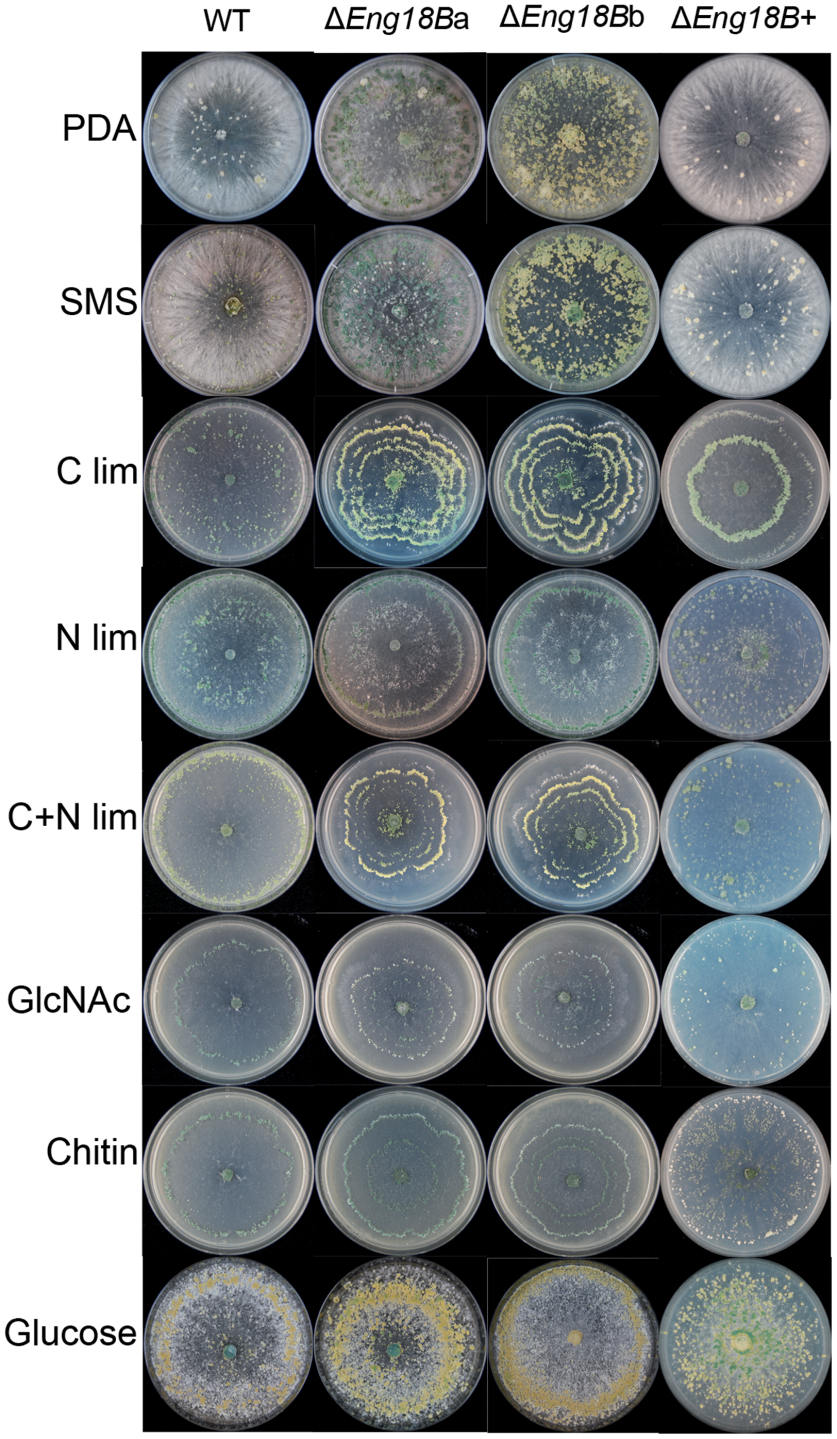
Colony morphology of WT, Δ*Eng18B* and Δ*Eng18B*+ *T*. *atroviride* strains in different nutrient regimes. *T*. *atroviride* strains were inoculated on solid PDA, SMS, C limitation, N limitation, C+N limitation, *N*-acetylglucosamine (GlcNAc), chitin and glucose medium. Photographs of representative plates were taken 7 days post inoculation after incubation at 25°C. The experiments were carried out in three biological replicates.

**Figure 4:**
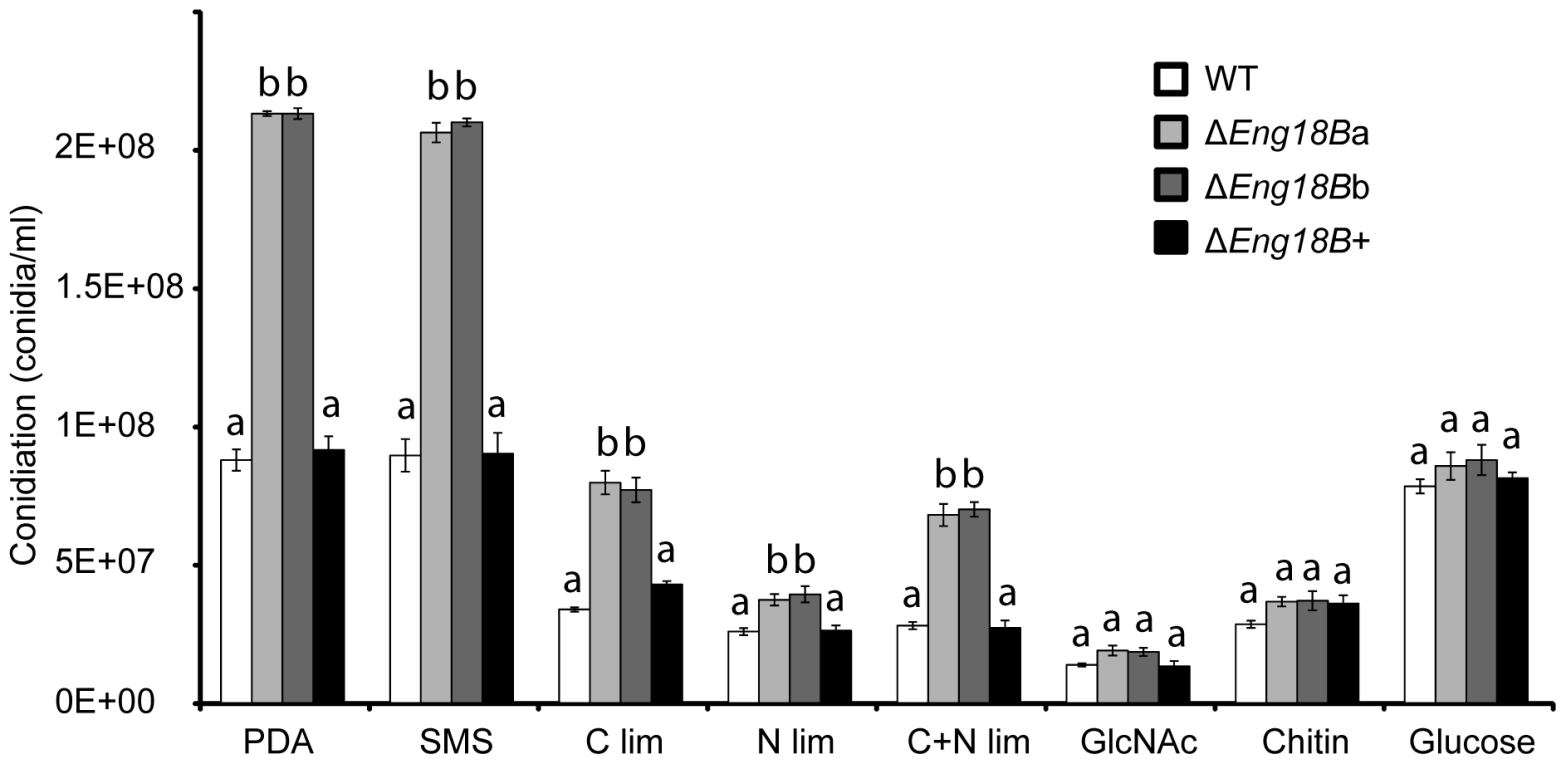
Conidiation of WT, Δ*Eng18B* and Δ*Eng18B*+ *T*. *atroviride* strains in different nutrient regimes. *T*. *atroviride* strains were inoculated on solid PDA, SMS, C limitation, N limitation, C+N limitation, *N*-acetylglucosamine (GlcNAc), chitin and glucose medium and incubated at 25°C in darkness for 7 days, with daily light exposures to induce conidiation. Conidial numbers were determined using a Bright-Line haemocytometer as per instruction of manufacturer. Error bars represent standard deviation based on three biological replicates. Different letters indicate statistical significance (*P*≤0.05) for strain differences within a single medium.

Differences in colony morphology between Δ*Eng18B* mutants and WT/Δ*Eng18B*+ strains were observed when they were grown on solid SMS medium supplemented with different nutritional sources (Fig. 3), especially with regard to patterns of conidiation. The Δ*Eng18B* mutants showed significantly increased conidiation in PDA, SMS, C lim, N lim and C+N lim medium (*P*≤0.018) compared to the WT and Δ*Eng18B*+ strains (Fig. 4). Microscopic investigation revealed no difference in the morphology of conidia, conidial germination rates or germ tube morphology between WT and Δ*Eng18B* mutants, neither in liquid PDB nor on PDA or SMS medium (Fig. S2).

### Disruption of *T*. *atroviride Eng18B* Results in Decreased Growth Rates

**Figure 5:**
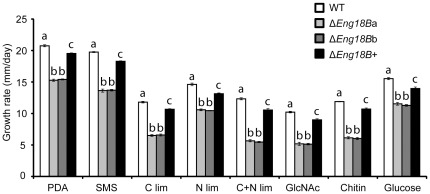
Growth rate of WT, Δ*Eng18B* and Δ*Eng18B*+ *T*. *atroviride* strains in different nutrient regimes. *T*. *atroviride* strains were inoculated on solid PDA, SMS, C limitation, N limitation, C+N limitation, *N*-acetylglucosamine (GlcNAc), chitin and glucose medium and incubated at 25°C in darkness. Growth rate was calculated from data recorded 3 days post inoculation. Error bars represent standard deviation based on three biological replicates. Different letters indicate statistical significance (*P*≤0.05) for strain differences within a single medium.

A significant reduction (*P*<0.001) in growth rates of Δ*Eng18B* strains was recorded when compared to the WT and Δ*Eng18B*+ strains under all culture conditions (Fig. 5). The maximum reduction was recorded in C+N lim medium (55%) followed by GlcNAc (49%) and colloidal chitin (49%). Furthermore, fungal biomass production was measured in liquid shake flask cultures, with equivalent composition to the previous experiment, but including RsCW. In contrast to growth on solid media, no significant differences were found in biomass production between Δ*Eng18B* strains and WT in liquid PDB, SMS, GlcNAc, RsCW, or C lim and N lim (*P*≥0.768). However, there was a significant (*P* = 0.004) reduction in biomass (42% of WT) in liquid SMS with colloidal chitin as sole carbon source. Microscopic investigation revealed no difference in hyphal morphology, including tip growth and hyphal branching patterns, between WT and Δ*Eng18B T*. *atroviride* strains either on PDA or solid SMS plates (data not shown).

### Disruption of *T*. *atroviride Eng18B* Results in Increased Resistance to Abiotic Stress

**Figure 6:**
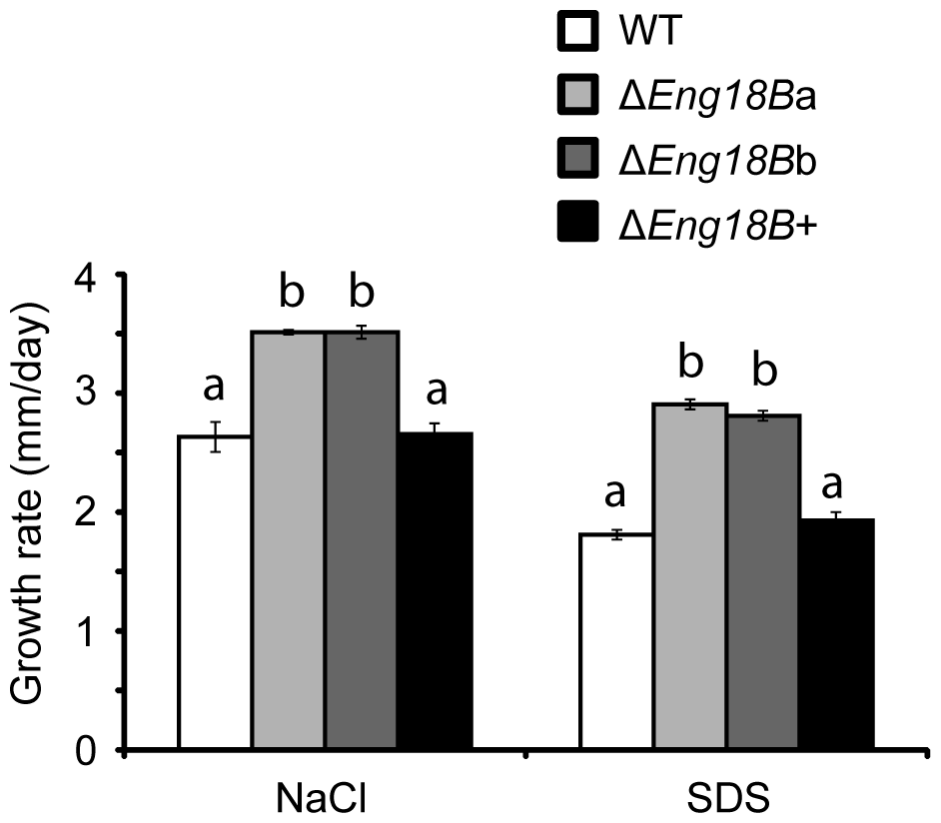
Growth rate of WT, Δ*Eng18B* and Δ*Eng18B*+ *T*. *atroviride* strains in abiotic stress medium. *T*. *atroviride* strains were inoculated on solid PDA plates supplemented with 1 M NaCl or 0.025% (w/v) SDS and incubated at 25°C in darkness. Growth rate was calculated from data recorded 7 days post inoculation. Error bars represent standard deviation based on three biological replicates. Different letters indicate statistical significance (*P*≤0.05) for strain differences within a single medium.

The potential role of *T*. *atroviride* Eng18B in influencing cell wall integrity was tested by growing WT, Δ*Eng18B* and Δ*Eng18B*+ *T*. *atroviride* strains on PDA plates supplemented with NaCl or SDS to induce cell wall stress. Growth rate of Δ*Eng18B* strains was found to be significantly increased by 25% on NaCl (*P*<0.001) and by 37% on SDS medium (*P*<0.001) compared with either WT or Δ*Eng18B*+ *T*. *atroviride* strains (Fig. 6). Fluorescence microscopy indicated no difference in cell wall chitin content between WT and Δ*Eng18B* strains (data not shown).

### Chitinase, NAGase and ENGase Enzyme Activities Remain Unaffected by *T*. *atroviride Eng18B* Disruption

*T*. *atroviride* WT and Δ*Eng18B* strains were grown in liquid SMS cultures supplemented with colloidal chitin or RsCW. Culture filtrates and mycelial homogenates were analyzed for NAGase or endochitinase activity, using 4MU-conjugated GlcNAc and tri-GlcNAc as substrates, respectively. No statistical difference (*P*≥0.721) in either enzyme activity was recorded between WT and *Eng18B* disruption *T*. *atroviride* strains, either in colloidal chitin or RsCW culture filtrates or mycelial homogenates.

*T*. *atroviride* WT and Δ*Eng18B* strains were grown in dextrose broth and ENGase deglycosylation activity was measured in culture filtrates and mycelial homogenates using an RNase B SDS polyacrylamide gel mobility shift assay. ENGase activity was detected by SDS-PAGE as a new band with lower molecular mass as compared to untreated RNase B in both culture filtrates and mycelial homogenates of WT and *Eng18B* disruption *T*. *atroviride* strains (Fig. S3). However, no difference in the ENGase activity in between WT and Δ*Eng18B* strains was detected.

### Disruption of *Eng18B* Reduces Antagonistic Ability Towards *B. Cinerea*

**Figure 7:**
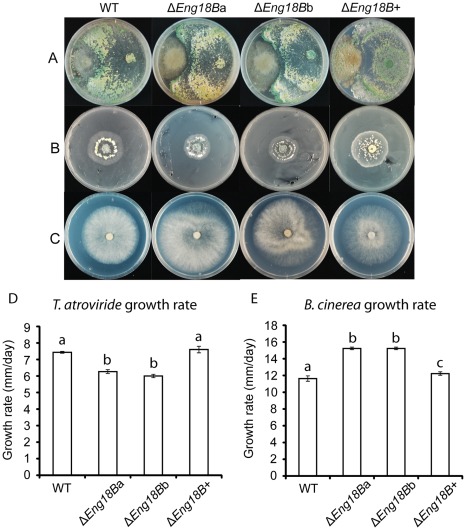
*In vitro* plate confrontation assays of WT, Δ*Eng18B* and Δ*Eng18B*+ *T*. *atroviride* strains. (A) Plate confrontation against *B*. *cinerea*. Agar plugs of *T*. *atroviride* (right side in the plate) and *B*. *cinerea* (left side in the plate) were inoculated on opposite sides in 9 cm SMS agar plates and incubated at 25°C in darkness. The experiment was performed in three replicates and photographs of representative plates were taken 15 days post inoculation. (B and D) Secretion assay of *B. cinerea*. Agar plugs of *B. cinerea* was inoculated on SMS agar plates covered with cellophane and incubated at 25°C in darkness. After reaching the same diameter the colony was removed together with the cellophane disc and the plates re-inoculated with a *T. atroviride* WT, Δ*Eng18B* or Δ*Eng18B*+ agar plug and incubated at 25°C in darkness. Growth rate was calculated from data recorded 5 days post inoculation. The experiment was performed in three replicates and photographs of representative plates were taken 5 days post inoculation. (C and E) Secretion assay of WT, Δ*Eng18B* and Δ*Eng18B*+ *T*. *atroviride* strains. Agar plugs of *T. atroviride* WT, Δ*Eng18B* or Δ*Eng18B*+ strains were inoculated on SMS agar plates covered with cellophane and incubated at 25°C in darkness. After reaching the same diameter the colony was removed together with the cellophane disc and the plates re-inoculated with a *B. cinerea* agar plug and incubated at 25°C in darkness. Growth rate was calculated from data recorded 5 days post inoculation. The experiment was performed in three replicates and photographs of representative plates were taken 5 days post inoculation. Different letters indicate statistically significant differences (*P*≤0.05) within experiments.

No difference in the ability of *T. atroviride* WT, Δ*Eng18B* and Δ*Eng18B*+ strains to overgrow and conidiate on *H. occidentale*, *P. chrysosporium*, *R. solani*, *A. nidulans*, *F. graminearum*, *N. crassa*, or *P. niederhauseri* was observed (data not shown). In contrast, Δ*Eng18B T*. *atroviride* strains failed to overgrow *B. cinerea* even after 30 days after contact while WT and Δ*Eng18B*+ *T*. *atroviride* strains overgrew *B. cinerea* in two days, completely lysing the *B*. *cinerea* mycelium (Fig. 7A). A secretion assay was used to further investigate the mechanism behind the reduced antagonistic ability. *T. atroviride* WT, Δ*Eng18B* and Δ*Eng18B*+ strains were grown on agar plates previously colonized by *B. cinerea* in order to test for sensitivity towards *B. cinerea* secreted factors. The growth rate of Δ*Eng18B T*. *atroviride* strains were 17% lower (*P*<0.001) than WT/Δ*Eng18B*+ after 5 days (Fig. 7B and D), which should be compared with the 26% growth rate reduction on PDA plates without *B. cinerea* pretreatment (Fig. 5). Furthermore, *B. cinerea* growth rate was significantly (*P*<0.001) higher on agar plates previously colonized by Δ*Eng18B T*. *atroviride* strains than on plates previously colonized by *T. atroviride* WT/Δ*Eng18B*+ strains (Fig. 7C and E).

## Discussion

The lack of signal peptide or GPI-anchor signal suggests a cytosolic localization of *T. atroviride* Eng18B, in contrast to the secreted ENGase Eng18A (homologous to *T. reesei* Eng18A (Endo T) [4]). The high sequence similarity between the catalytic modules of *T*. *atroviride* Eng18A and Eng18B (50% identical aa) indicates ENGase activity for Eng18B. As a comparison, the catalytic modules of the three *T. reesei* B1/B2-group endochitinases Chi18–13, Chi18–16 and Chi18–17 [40], display 35–42% aa identity while the catalytic modules of the *T. reesei* endochitinase Chi18–13 and the A5-group exochitinase Chi18–5 [40] display only 20% sequence identity. However, deletion of *T*. *atroviride Eng18B* does not reduce measurable ENGase enzyme activity, which is probably due to high ENGase activity of the fungal ENGase Eng18A that is still intact in the *T*. *atroviride Eng18B* deletion mutants. Compensatory effects from paralogous proteins are a common problem when trying to deduce functional contributions of individual isozymes. Homology modelling studies of *T. atroviride* Eng18B (based on the structure of *T. reesei* Eng18A) indicate that *T*. *atroviride* Eng18B has an active site architecture that is consistent with ENGase cleavage of the bond between the two β-1,4-linked GlcNAc units that connect glycoproteins to their linked oligosaccharide chains. The active site of *T*. *atroviride* Eng18B is highly conserved compared with the active site of *T. reesei* Eng18A. Regions that display high amino-acid variation between *Trichoderma* Eng18B orthologs are found to be located distal from the catalytic cleft, which suggests conserved substrate specificity between the orthologs.

Bacterial ENGases are shown to remove the oligosaccharide-protective coat from foreign glycoproteins in order to provide peptides for nutritional purposes [41]. Although this function may be possible for the secreted *T*. *atroviride* Eng18A orthologs [4], it is less likely for the putatively intracellular *T*. *atroviride* Eng18B. Instead, intracellular ENGase activity is shown to be part of ERAD-degradation of misfolded glycoproteins [9], [10]. More specifically, *N*-glycan carbohydrate chains are removed from glycoproteins by PNGases to generate fOS-GN2, followed by further degradation by ENGases to fOS-GN1 and α-mannosidases before transport into the lysosome. The situation is further complicated in filamentous fungi where the intracellular PNGase apparently carries amino acid substitutions that abolish PNGase activity [14]. We may speculate that disruption of the ERAD ENGase activity interferes with *N*-glycan-dependent aspects of glycoprotein maturation and secretion such as clogging of the secretory pathway or secretion of misfolded and/or erroneously glycosylated proteins.

We started the functional characterization of *T. atroviride* Eng18B by investigating the regulatory patterns of *Eng18B*. The gene is expressed in all tested conditions, which is expected for a function in ERAD-degradation of misfolded glycoproteins. Gene transcription is induced during interactions with *R. solani* and *B. cinerea*, which can be explained by increased secretion of cell wall degrading enzymes during the mycoparasitic attack, accompanied by an increase in the amount of misfolded glycoproteins. Interestingly, *T*. *atroviride Eng18B* gene expression is repressed by GlcNAc, which merit further discussion. Studies in rats show that the cytosol to lysosome transport of fOS-GN1 is blocked by GlcNAc and other chitooligosaccharides [42]. The repression of *T*. *atroviride Eng18B* by GlcNAc may thus represent a mechanism to adjust upstream steps in the ERAD-degradation pathway to the block in fOS-GN1 lysosome import. Furthermore, *T*. *atroviride Eng18B* is not regulated by glucose repression like many *Trichoderma* carbohydrate-degrading enzymes such as cellulases [43]. The absence of glucose repression is also observed for the secreted *T. reesei* ENGase Eng18A, for which expression and activity is not co-regulated with cellulolytic activities [4], [44].

There is no difference in biomass production in liquid cultures between Δ*Eng18B* and WT strains of *T. atroviride*, with the exception of cultures where colloidal chitin constitutes the only source of carbon. Chitin was used in the current study as an example substrate for secreted hydrolytic enzymes. The chitin utilization defect establishes a link between *T. atroviride* Eng18B function and chitin degradation, possibly by impaired secretion or suboptimal activity of certain secreted chitinolytic enzymes due to defective folding or glycosylation [45], or by other as yet unidentified *T. atroviride* enzymes involved in chitin catabolism. Reduced biomass production is not observed when *R. solani* cell wall material is used as a carbon source for *T. atroviride* cultures, which can be attributed to the presence of other components of the cell wall such as β-glucans and proteins that can be utilized as nutrients. Paradoxically, no reduction of total NAGase or endochitinase activities is observed in the *T. atroviride Eng18B* disruption strains that would explain the lower ability of the fungus to utilize chitin as a nutrient source. However, chitin is a complex substrate that requires the concerted action of several endo- and exo-acting chitinolytic enzymes, while NAGase and endochitinase activities in the current study were measured using 4MU-conjugated GlcNAc and tri-GlcNAc which are structurally more simple substrates.

Disruption of the *T*. *atroviride Eng18B* gene results in several phenotypic effects related to growth and development in *T*. *atroviride*, including lower growth rates on normal media, higher growth rates under conditions of cell wall stress, and increased or alternatively earlier conidiation compared to the WT strain under similar conditions. The common denominator for these phenotypes may be impaired cell wall function. The fungal cell wall is a highly dynamic structure that changes continuously during different stages of the life cycle and in response to different environmental conditions [39], [46]. The model of cell wall synthesis and remodelling include a balance between chitin synthesis for adequate strength to protect cells under adverse environmental conditions and chitin hydrolysis to provide sufficient plasticity to the cell wall for growth and morphogenesis [47], [48]. Although no increase of cell wall chitin content is measured in *T*. *atroviride* Δ*Eng18B* strains, cell wall structure may still be different due to the observed chitin degradation defect, consequently leading to a reduced growth rate on solid media. A previous study showed that disruption of the intracellular PNG-1 in *N. crassa* results in a growth defect associated with the hyphal tip [13]. However, the swollen-tip phenotype observed for *N. crassa* in that study is not observed for the *T. atroviride* Δ*Eng18B* strains in the current study, but emphasizes the connection between ERAD protein degradation and hyphal tip growth.

According to this idea, Δ*Eng18B T*. *atroviride* strains may have a more rigid cell wall structure that consequently leads to better resistance against cell wall stress. Therefore, Δ*Eng18B* and WT/Δ*Eng18B*+ *T*. *atroviride* strains were exposed to chemicals used to test cell wall integrity on solid media. Our data confirm that Δ*Eng18B* strains grow faster than the WT/Δ*Eng18B*+ strains under conditions of cell wall stress, which is in line with the idea of a more rigid cell wall structure. *S. cerevisiae* cells under environmental stress can change the relative amount of their cell wall polymers, where increased levels of chitin results in a reinforced cell wall [46].

Several studies in *N. crassa* and *A. nidulans* illustrate a trade-off between hyphal growth and conidiation; deletion of genes involved in cAMP dependent G-protein signalling reduces filamentous growth but causes premature conidiation [49], [50]. In the current study, the reduced growth rate of Δ*Eng18B T*. *atroviride* strains are accompanied by enhanced or premature conidiation. A connection between chitin ring deposition and temporal and spatial bud emergence for conidiation is reported in *S*. *cerevisiae* [46]. However, loss of *T*. *atroviride* Eng18B function in the fungus does not affect conidial morphology or germination, germ-tube development, hyphal morphology or branching.

Our study shows that Eng18B is necessary for the antagonistic ability of *T. atroviride* against *B. cinerea* in plate confrontation assays, but not against any of the other tested fungi or oomycete. In addition, the fact that *B. cinerea* grow less well on plates previously colonized by WT/Δ*Eng18B*+ *T*. *atroviride* strains than by the Δ*Eng18B T*. *atroviride* strains establish that the reduced antagonistic ability can at least partly be attributed to a secreted factor. This may be due to impaired secretion or defective folding or glycosylation of certain secreted hydrolytic enzymes that are specifically important for the mycoparasitic attack on *B. cinerea*. Another component in the reduced antagonistic ability of Δ*Eng18B T*. *atroviride* strains may involve a greater sensitivity towards secreted *B. cinerea* toxins or enzymes due to impaired cell wall function. However, our data show that this is not the case; the growth rate reduction of *Eng18B* disruption strains on PDA plates previously colonized by *B. cinerea* is smaller than the reduction on normal PDA. This can be explained by the higher resistance to abiotic stress that is attributed to the Δ*Eng18B* strains and that *B. cinerea* secretes hydrolytic enzymes and toxins into the PDA plates.

The present study constitutes the first study of the biological role of a fungal member from the novel GH family 18 B5 ENGase subgroup, *T. atroviride* Eng18B, with focus on its role in fungal growth and development. We show that *T. atroviride* Eng18B is involved in hyphal growth, tolerance to abiotic stress, conidiation, chitin utilization and the antagonistic ability of *T. atroviride* towards *B. cinerea*. The exact mechanistic relationships between *T*. *atroviride* Eng18B function and the observed phenotypic effects require further investigation. The *T. atroviride* Δ*Eng18B* strains generated in the current study will be a valuable tool to further dissect the ERAD-pathway dependent protein degradation in filamentous fungi.

## Supporting Information

Figure S1 **Schematic representation of disruption cassette and characterization of Δ** ***Eng18B*** **mutant** ***T*** **.** ***atroviride*** **strains using PCR and RT-PCR.** (A) Organisation of *Eng18B* locus in WT and mutant strain of *T. atroviride*. The *Eng18B* coding region was replaced by *hph* cassette by homologous recombination resulting in generation of Δ*Eng18B* mutants. The small arrowheads indicate the location of primers used to construct the disruption cassette and analysis of mutants using PCR. The large arrowheads indicate the size of amplified PCR products. Abbreviations: LB, left boarder; RB, right boarder. Characterization of Δ*Eng18B* mutant *T*. *atroviride* strains using PCR and RT-PCR. (B) PCR verification of *hph* cassette (1.5 kb) from genomic DNA of putative transformants and WT strains using specific primer pair (P3/P4). M, gene ruler DNA ladder mix; 1–9, nine independent Δ*Eng18B* mutants; 10, disruption vector (pPm43GW-Eng18B-ko) as positive control; and 11–12, WT. (C & D) PCR verification using primers located in the *hph* gene (P3/P4) in combination with primers located upstream and downstream from the disruption cassette (P11/P12). PCR products of 2.8 kb and 3.1 kb using primers P4/P11 and P3/P12 were expected from a correct gene replacement. M, gene ruler DNA ladder mix; 1–10, independent Δ*Eng18B* mutants; 11, WT; and 12, water control. (E) PCR verification of Δ*Eng18B* mutants using primer pair (P11/P12) flanking the disruption cassette. PCR products of 4.3 kb and 3.8 kb were expected from the mutant and WT strains, respectively. M, gene ruler DNA ladder mix; 1–10, independent Δ*Eng18B* mutants; 11, WT; and 12 water control. (F) RT-PCR analysis of gene expression in mutant and WT strains, using *Eng18B* and *hph* specific primers P19/P20 and P13/P14, respectively. Housekeeping gene *tef1* was used as internal control of cDNA quality and amplified by P7/P8 primers. M, gene ruler DNA ladder mix; 1–4, independent Δ*Eng18B* mutant strains; and 5, WT. Primer combinations used for PCR and RT-PCR are given above the images. (G) PCR verification of *nat1* cassette from genomic DNA of putative transformants and WT strains using specific primer pair (P33/P34). M, gene ruler DNA ladder mix; 1–6, six independent Δ*Eng18B*+ complemented strains; and 7, WT. (H) RT-PCR analysis of *Eng18B* expression in WT, Δ*Eng18B* knock-out and Δ*Eng18B*+ complemented strains, using *Eng18B* specific primers P19/P20. M, gene ruler DNA ladder mix; 1, WT; 2–3, independent Δ*Eng18B* knock-out strains; and 4–6, independent Δ*Eng18B*+ complemented strains.
(PDF)

Figure S2 **Germ tube morphology of WT and Δ** ***Eng18B T*** **.** ***atroviride*** **strains.** Conidia were inoculated in PDB medium and monitored using a Zeiss Axioplan microscope equipped with Leica application suite version 3.6.0. Images were taken 20 h post inoculation using a Leica DFC295 digital camera at the same magnification.
(PDF)

Figure S3 **SDS-PAGE analysis of ENGase-type activity in** ***T*** **.** ***atroviride*** **using (A) culture filtrate or (B) cytosolic fraction.** *T. atroviride* WT and Δ*Eng18B* mutants were grown in dextrose broth for 48 h at 25°C. Forty µl culture filtrate or cytosolic fraction was mixed with 100 µg of RNase B and incubated at room temperature for 24 h for deglycosylation. Twenty µl of the reactions were mixed with 5 µl of loading dye and heat denatured at 100°C for 10 min before loading. L, protein ladder; 1, RNAse B incubated with dextrose broth; 2, fresh RNAse B; 3, WT culture filtrate or cytosolic fraction incubated with RNAse B; 4 and 5, Δ*Eng18B* mutants culture filtrate or cytosolic fraction incubated with RNAse B; 6, WT culture filtrate or cytosolic fraction without RNAse B; 7 and 8, Δ*Eng18B* mutants culture filtrate or cytosolic fractions without RNAse B.
(PDF)

Table S1 **Primers used in the current study.** ^a^attB and attBr sequences for multisite gateway BP recombination are underlined.
(DOCX)
