# Supplementary material for: Disruption of the Eng18B ENGase Gene in the Fungal Biocontrol Agent Trichoderma atroviride Affects Growth, Conidiation and Antagonistic Ability
Source: PLoS One. 2012 May 7;7(5):e36152. doi: 10.1371/journal.pone.0036152 (PMC3346758; doi:10.1371/journal.pone.0036152)
Supplement: Figure S1 — Schematic representation of disruption cassette and characterization of Δ Eng18B mutant T . atroviride strains using PCR and RT-PCR. (A) Organisation of Eng18B locus in WT and mutant strain of T. atroviride. The Eng18B coding region was replaced by hph cassette by homologous recombination resulting in generation of ΔEng18B mutants. The small arrowheads indicate the location of primers used to construct the disruption cassette and analysis of mutants using PCR. The large arrowheads indicate the size of amplified PCR products. Abbreviations: LB, left boarder; RB, right boarder. Characterization of ΔEng18B mutant T. atroviride strains using PCR and RT-PCR. (B) PCR verification of hph cassette (1.5 kb) from genomic DNA of putative transformants and WT strains using specific primer pair (P3/P4). M, gene ruler DNA ladder mix; 1–9, nine independent ΔEng18B mutants; 10, disruption vector (pPm43GW-Eng18B-ko) as positive control; and 11–12, WT. (C & D) PCR verification using primers located in the hph gene (P3/P4) in combination with primers located upstream and downstream from the disruption cassette (P11/P12). PCR products of 2.8 kb and 3.1 kb using primers P4/P11 and P3/P12 were expected from a correct gene replacement. M, gene ruler DNA ladder mix; 1–10, independent ΔEng18B mutants; 11, WT; and 12, water control. (E) PCR verification of ΔEng18B mutants using primer pair (P11/P12) flanking the disruption cassette. PCR products of 4.3 kb and 3.8 kb were expected from the mutant and WT strains, respectively. M, gene ruler DNA ladder mix; 1–10, independent ΔEng18B mutants; 11, WT; and 12 water control. (F) RT-PCR analysis of gene expression in mutant and WT strains, using Eng18B and hph specific primers P19/P20 and P13/P14, respectively. Housekeeping gene tef1 was used as internal control of cDNA quality and amplified by P7/P8 primers. M, gene ruler DNA ladder mix; 1–4, independent ΔEng18B mutant strains; and 5, WT. Primer combinations used for PCR and RT-PCR are given above [file pone.0036152.s001.pdf]

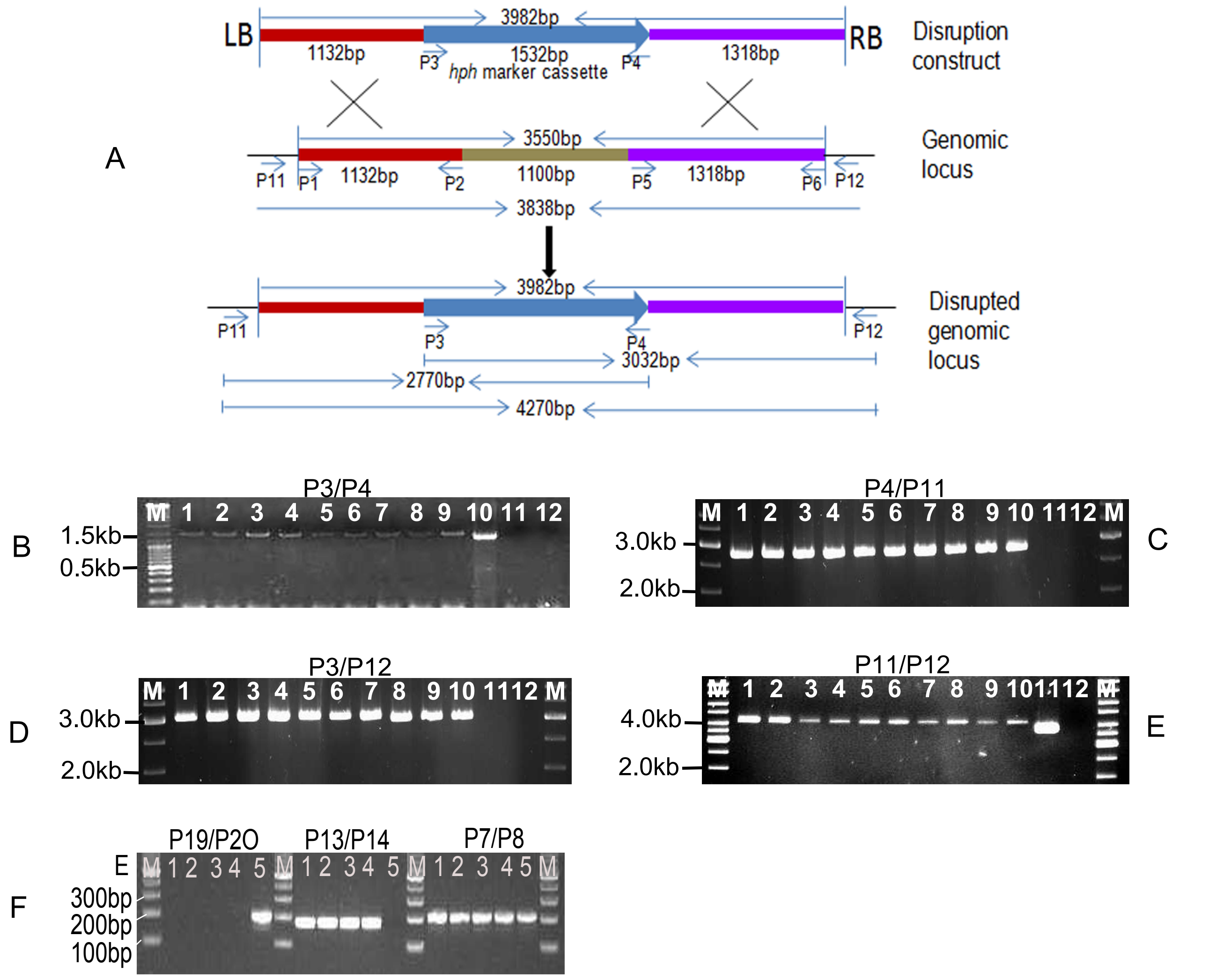

Figure S1. Schematic representation of disruption cassette and characterization of Δ *Eng18B* mutant strains using PCR and RT-PCR. (A) Organisation of *Eng18B* locus in WT and mutant strain of *T. atroviride*. The *Eng18B* coding region was replaced by *hph* cassette by homologous recombination resulting in generation of Δ*Eng18B* mutants. The small arrow heads indicate the location of primers used to construct the disruption cassette and analysis of mutants using PCR. The large arrow heads indicate the size of amplified PCR products. Abbreviations: LB, left boarder; RB, right boarder. Characterization of Δ*Eng18B* mutant strains using PCR and RT-PCR. (B) PCR verification of *hph* cassette (1.5 kb) from genomic DNA of putative transformants and WT strains using specific primer pair (P3/P4). M, gene ruler DNA ladder mix; 1-9, nine independent Δ*Eng18B* mutants; 10, disruption vector (pPm43GW-Eng18B-ko) as positive control; and 11-12, WT. (C & D) PCR verification using primers located in the *hph* gene (P3/P4) in combination with primers located upstream and downstream from the disruption cassette (P11/P12). PCR products of 2.8 kb and 3.1 kb using primers P4/P11 and P3/P12 were expected from a correct gene replacement. M, gene ruler DNA ladder mix; 1-10, independent Δ*Eng18B* mutants; 11, WT; and 12, water control. (E) PCR verification of Δ*Eng18B* mutants using primer pair (P11/P12) flanking the disruption cassette. PCR products of 4.3 kb and 3.8 kb were expected from the mutant and WT strains, respectively. M, gene ruler DNA ladder mix; 1-10, independent Δ*Eng18B* mutants; 11, WT; and 12 water control. (F) RT-PCR analysis of gene expression in mutant and WT strains, using *Eng18B* and *hph* specific primers P19/P20 and P13/P14, respectively. Housekeeping gene *tef1* was used as internal control of cDNA quality and amplified by P7/P8 primers. M, gene ruler DNA ladder mix; 1-4, independent Δ*Eng18B* mutant strains; and 5, WT. Primer combinations used for PCR and RT-PCR are given above the images.

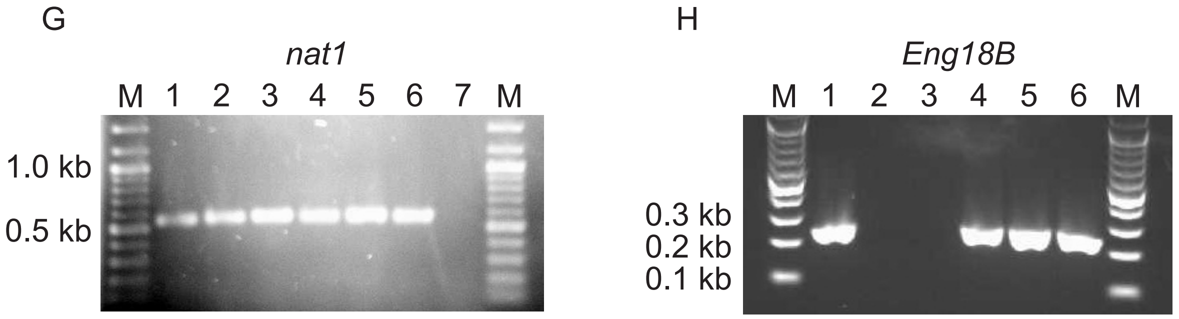

FIG S1 continued. Characterization of Δ*Eng18B*+ complementation strains. (G) PCR verification of *nat1* cassette from genomic DNA of putative transformants and WT strains using specific primer pair (P33/P34). M, gene ruler DNA ladder mix; 1-6, six independent Δ*Eng18B*+ complemented strains; and 7, WT. (H) RT-PCR analysis of *Eng18B* expression in WT, Δ*Eng18B* knock-out and Δ*Eng18B*+ complemented strains, using *Eng18B* specific primers P19/P20. M, gene ruler DNA ladder mix; 1, WT; 2-3, independent Δ*Eng18B* knock-out strains; and 4-6, independent Δ*Eng18B*+ complemented strains.
