# Supplementary material for: Disruption of the Eng18B ENGase Gene in the Fungal Biocontrol Agent Trichoderma atroviride Affects Growth, Conidiation and Antagonistic Ability
Source: PLoS One. 2012 May 7;7(5):e36152. doi: 10.1371/journal.pone.0036152 (PMC3346758; doi:10.1371/journal.pone.0036152)
Supplement: Figure S2 — Germ tube morphology of WT and Δ Eng18B T . atroviride strains. Conidia were inoculated in PDB medium and monitored using a Zeiss Axioplan microscope equipped with Leica application suite version 3.6.0. Images were taken 20 h post inoculation using a Leica DFC295 digital camera at the same magnification. (PDF) [file pone.0036152.s002.pdf]

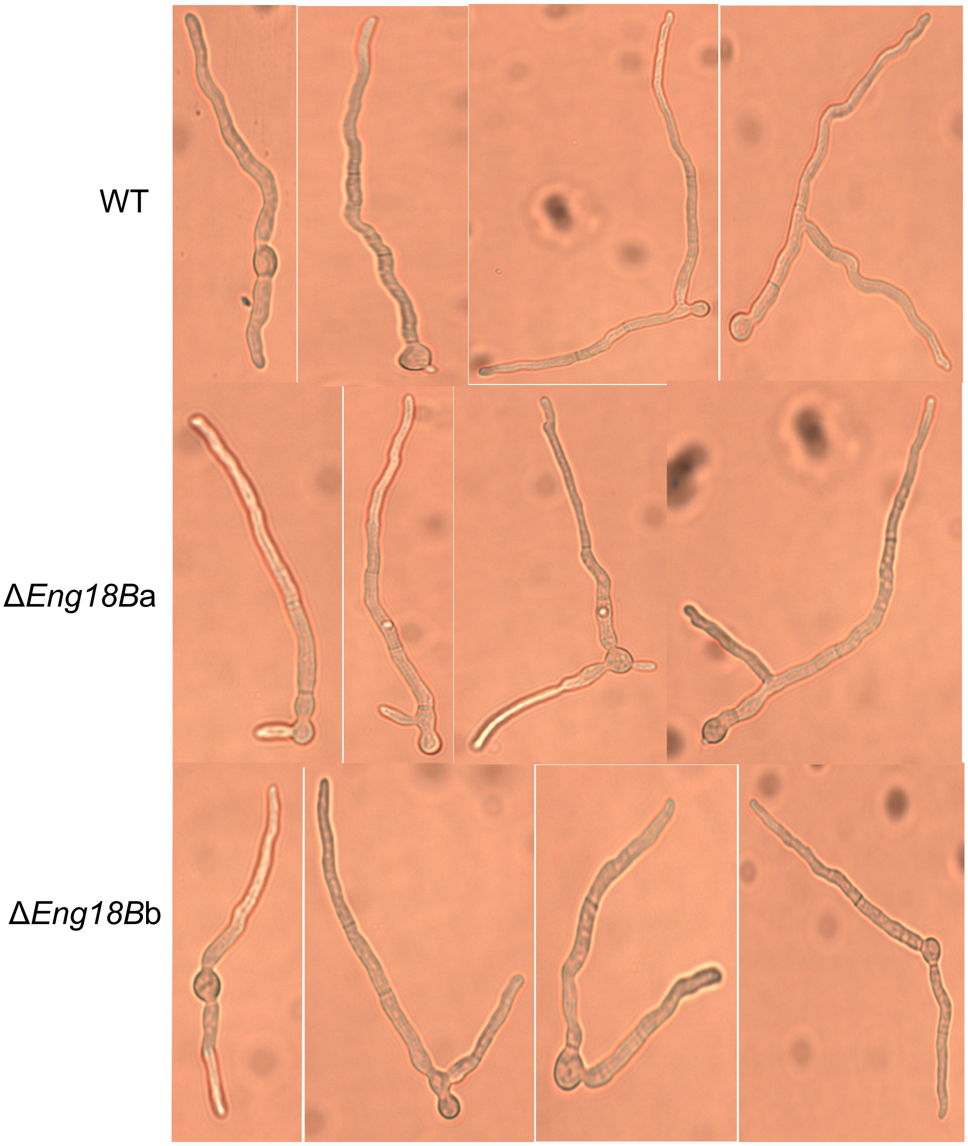

Figure S2. Germ tube morphology of WT and Δ*Eng18B* strains. Conidia were inoculated in PDB medium and monitored using a Zeiss Axioplan microscope equipped with Leica application suite version 3.6.0. Images were taken 20 h post inoculation using a Leica DFC295 digital camera at the same magnification.
