# Supplementary material for: Disruption of the Eng18B ENGase Gene in the Fungal Biocontrol Agent Trichoderma atroviride Affects Growth, Conidiation and Antagonistic Ability
Source: PLoS One. 2012 May 7;7(5):e36152. doi: 10.1371/journal.pone.0036152 (PMC3346758; doi:10.1371/journal.pone.0036152)
Supplement: Figure S3 — SDS-PAGE analysis of ENGase-type activity in T . atroviride using (A) culture filtrate or (B) cytosolic fraction. T. atroviride WT and ΔEng18B mutants were grown in dextrose broth for 48 h at 25°C. Forty µl culture filtrate or cytosolic fraction was mixed with 100 µg of RNase B and incubated at room temperature for 24 h for deglycosylation. Twenty µl of the reactions were mixed with 5 µl of loading dye and heat denatured at 100°C for 10 min before loading. L, protein ladder; 1, RNAse B incubated with dextrose broth; 2, fresh RNAse B; 3, WT culture filtrate or cytosolic fraction incubated with RNAse B; 4 and 5, ΔEng18B mutants culture filtrate or cytosolic fraction incubated with RNAse B; 6, WT culture filtrate or cytosolic fraction without RNAse B; 7 and 8, ΔEng18B mutants culture filtrate or cytosolic fractions without RNAse B. (PDF) [file pone.0036152.s003.pdf]

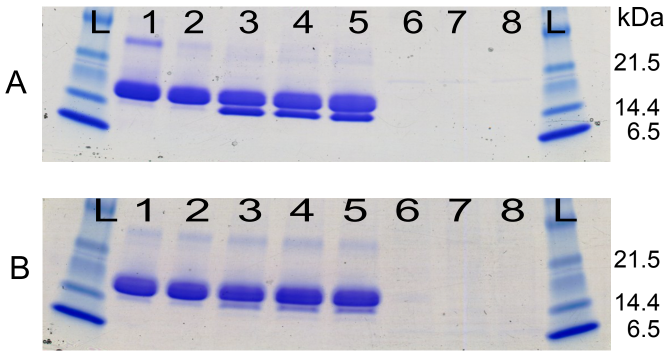

Figure S3. SDS-PAGE analysis of ENGase-type activity in *T. atroviride* using (A) culture filtrate or (B) cytosolic fraction. *T. atroviride* WT and Δ *Eng18B* mutants were grown in dextrose broth for 48h at 25°C. Forty µl culture filtrate or cytosolic fraction was mixed with 100 µg of RNase B and incubated at room temperature for 24h for deglycosylation. Twenty µl of the reactions were mixed with 5 µl of loading dye and heat denatured at 100°C for 10 min before loading. L, protein ladder; 1, RNAse B incubated with dextrose broth; 2, fresh RNAse B; 3, WT culture filtrate or cytosolic fraction incubated with RNAse B; 4 and 5, Δ*Eng18B* mutants culture filtrate or cytosolic fraction incubated with RNAse B; 6, WT culture filtrate or cytosolic fraction without RNAse B; 7 and 8, Δ *Eng18B* mutants culture filtrate or cytosolic fractions without RNAse B.
