# Supplementary material for: Disruption of the Eng18B ENGase Gene in the Fungal Biocontrol Agent Trichoderma atroviride Affects Growth, Conidiation and Antagonistic Ability
Source: PLoS One. 2012 May 7;7(5):e36152. doi: 10.1371/journal.pone.0036152 (PMC3346758; doi:10.1371/journal.pone.0036152)
Supplement: Table S1 — Primers used in the current study. aattB and attBr sequences for multisite gateway BP recombination are underlined. (DOCX) [file pone.0036152.s004.docx]

TABLE S1: Primers used in the current study

| Name | Target gene | Sequence (5´ → 3`)^a^ |
| --- | --- | --- |

| P1 | *Eng18B* | GGGG ACA ACT TTG TAT AGA AAA GTT G: TTT ATT GAC CGT AGG GGC TCT GA |
| --- | --- | --- |
| P2 | *Eng18B* | GGGG AC TGC TTT TTT GTA CAA ACT TG: GCA CCC CGC TAT AAC TGA CG |
| P3 | *hph* | GGGG ACA AGT TTG TAC AAA AAA GCA GGC T: GCG CGC AAT TAA CCC TCA C |
| P4 | *hph* | GGGG AC CAC TTT GTA CAA GAA AGC TGG GT: GAA TTG CGC GTA CAG AAC TCC |
| P5 | *Eng18B* | GGGG ACA GCT TTC TTG TAC AAA GTG G: ACG ACA AGC CGA TAC AAA CTC CT |
| P6 | *Eng18B* | GGGG AC AAC TTT GTA TAA TAA AGT TG: CAA CTG CAA CCA TGC CTA ACG |
| P7 | *tef1* | TCC GGC AAG TCT ACC ACC AC |
| P8 | *tef1* | CGA ACT TCC AGA GGG CAA TG |
| P9 | *Eng18B* | AAA GCC GAG TGT GCG AGA TT |
| P10 | *Eng18B* | AGA TGA CTG AGG AGG ATG GAG AG |
| P11 | *Eng18B* | TGT CGT GGG CAG CGG AGA AA |
| P12 | *Eng18B* | CAT CCT CTG TGA CTG TGG CAT TGG |
| P13 | *hph* | ACG GCG GGA GAT GCA ATA GGT |
| P14 | *hph* | GCT TCG ATG TAG GAG GGC GTG G |
| P15 | Contig 15 | GGGG ACA GCT TTC TTG TAC AAA GTG G: CGG TGG GTG GTG ATG AGT TA |
| P16 | Contig 15 | GGGG ACA ACT TTG TAT AAT AAA GTT G: CAA CGG GAT GAC GAA TGT AAC |
| P17 | Actin | CTC ACA TCC TTC GCC AAT CAC TC |
| P18 | Actin | AGC CCA GCT GCC ATA CAC AAG |
| P19 | *Eng18B* | GTA ATA TAA TCC GTG CAA GG |
| P20 | *Eng18B* | TGC GAT TTC CTT GCC GCG CAT |
| P21 | *Eng18B* | TTC AAC CGG CCT CCA ATC C |
| P22 | *Eng18B* | GAC CTC CAG GGC CTC GTA ATC |
| P23 | *Eng18B* | TGG GGT TAA AGT TTT GGG TAT GCT |
| P24 | *Eng18B* | TCC ACC AGG AAG GCT ATT GAA GT |
| P25 | *Eng18B* | CGT TGC TGC TGC CCT CCT G |
| P26 | *Eng18B* | GAA TTG GCT AGG AGT TTG TAT CGG C |
| P29 | *Eng18B* | GGGG ACA GCT TTC TTG TAC AAA GTG G: TTT ATT GAC CGT AGG GGC TCT GA |
| P30 | *Eng18B* | GGGG AC AAC TTT GTA TAA TAA AGT TG: CAA CTG CAA CCA TGC CTA ACG |
| P31 | *nat1* | GGGG ACA AGT TTG TAC AAA AAA GCA GGC TTA: CAG AAT TCG TGA TGA ATT |
| P32 | *nat1* | GGGG AC CAC TTT GTA CAA GAA AGC TGG GTA: GAC GAA TTC AGA TGG GCC |
| P33 | *nat1* | GGC TGG AGC TAG TGG AGG TCA ACA |
| P34 | *nat1* | GTG CTC CGG GGC GAC CTC |

^a^attB and attBr sequences for multisite gateway BP recombination are underlined.
